# Possible effects of *EXT2* on mesenchymal differentiation - lessons from the zebrafish

**DOI:** 10.1186/1750-1172-9-35

**Published:** 2014-03-14

**Authors:** Malgorzata I Wiweger, Carlos E de Andrea, Karel W F Scheepstra, Zhe Zhao, Pancras C W Hogendoorn

**Affiliations:** 1 Department of Pathology, Leiden University Medical Center, Leiden, The Netherlands; 2 Current address: Zebrafish Core Facility, International Institute of Molecular and Cell Biology, Warsaw, Poland; 3 Department of Histology and Pathology, University of Navarra, Pamplona, Spain; 4 Nuffield Department of Medicine, Ludwig Institute for Cancer Research, University of Oxford, Oxford, UK

**Keywords:** Zebrafish, Heparan sulphate, Bone, Fat, Osteochondroma, Exostosis, MHE/HME, Osteoblasts, Differentiation, Bone tumour

## Abstract

**Background:**

Mutations in the *EXT* genes disrupt polymerisation of heparan sulphates (HS) and lead to the development of osteochondroma, an isolated/sporadic- or a multifocal/hereditary cartilaginous bone tumour. Zebrafish (*Danio rerio*) is a very powerful animal model which has shown to present the same cartilage phenotype that is commonly seen in mice model and patients with the rare hereditary syndrome, Multiple Osteochondroma (MO).

**Methods:**

Zebrafish *dackel (dak)* mutant that carries a nonsense mutation in the *ext2* gene was used in this study. A panel of molecular, morphological and biochemical analyses was used to assess at what step bone formation is affected and what mechanisms underlie changes in the bone formation in the *ext2* mutant.

**Results:**

During bone development in the *ext2*^*-/-*^ zebrafish, chondrocytes fail to undergo terminal differentiation; and pre-osteoblasts do not differentiate toward osteoblasts. This inadequate osteogenesis coincides with increased deposition of lipids/fats along/in the vessels and premature adipocyte differentiation as shown by biochemical and molecular markers. Also, the *ext2*-null fish have a muscle phenotype, i.e. muscles are shorter and thicker. These changes coexist with misshapen bones. Normal expression of *runx2* together with impaired expression of *osterix* and its master regulator - *xbp1* suggest that unfolded protein responses might play a role in MO pathogenesis.

**Conclusions:**

Heparan sulphates are required for terminal differentiation of the cartilaginous template and consecutive formation of a scaffold that is needed for further bone development. HS are also needed for mesenchymal cell differentiation. At least one copy of *ext2* is needed to maintain the balance between bone and fat lineages, but homozygous loss of the *ext2* function leads to an imbalance between cartilage, bone and fat lineages. Normal expression of *runx2* and impaired expression of *osterix* in the *ext2*^*-/-*^ fish indicate that HS are required by osteoblast precursors for their further differentiation towards osteoblastic lineage. Lower expression of *xbp1,* a master regulator of *osterix,* suggests that HS affect the ‘unfolded protein response’, a pathway that is known to control bone formation and lipid metabolism. Our observations in the *ext2*-null fish might explain the musculoskeletal defects that are often observed in MO patients.

## Introduction

Bone formation and homeostasis are complex processes in which many cell types and various signalling pathways are involved. Chondrocytes and osteoblasts originate from the same precursors - mesenchymal stem cells, which can also differentiate towards adipocytes, fibroblasts, myoblasts and epithelial cells. The osteoblast lineage is under strict control of *RUNT-RELATED TRANSCRIPTION FACTOR 2 (RUNX2)* and its downstream target - *SP7*/*OSTERIX,* which can work in a *Runx-*dependent and/or an independent manner. *Runx2* and *Osterix* mRNA are expressed in the immature chondro/osteoprogenitor cells and osteoblasts only [1] and alterations in the expression of either of the two genes affect bone development. *Runx*- and *Osterix*-null mice have normal cartilage but their reduced or absent expression of type I collagen, bone sialoprotein, osteonectin, osteopontin, and osteocalcin indicate that bone development is blocked at the step of pre-osteoblast to osteoblast differentiation [2]. Mammalian cells treated with an *Osterix* inhibitor, dexamethasone, enter an adipogenic- instead of osteoblastic lineage [3]. This imbalance between bone and fat is a known phenomenon. For example, it has been shown that knockout mice, which are heterozygous for *Peroxisome proliferator-activated receptor γ(PPARγ* have impaired adipogenesis, coinciding with an increased osteoblast number [4]. Other signalling molecules such as wingless (Wnt), bone morphogenic protein (BMP), and hedgehog were also shown to trigger the switch between different lineages including a bone-to-fat change. Remarkably, in all of these pathways, receptor-ligand binding and gradient formation is dependent on heparan sulphates (HS).

**Figure 1:**
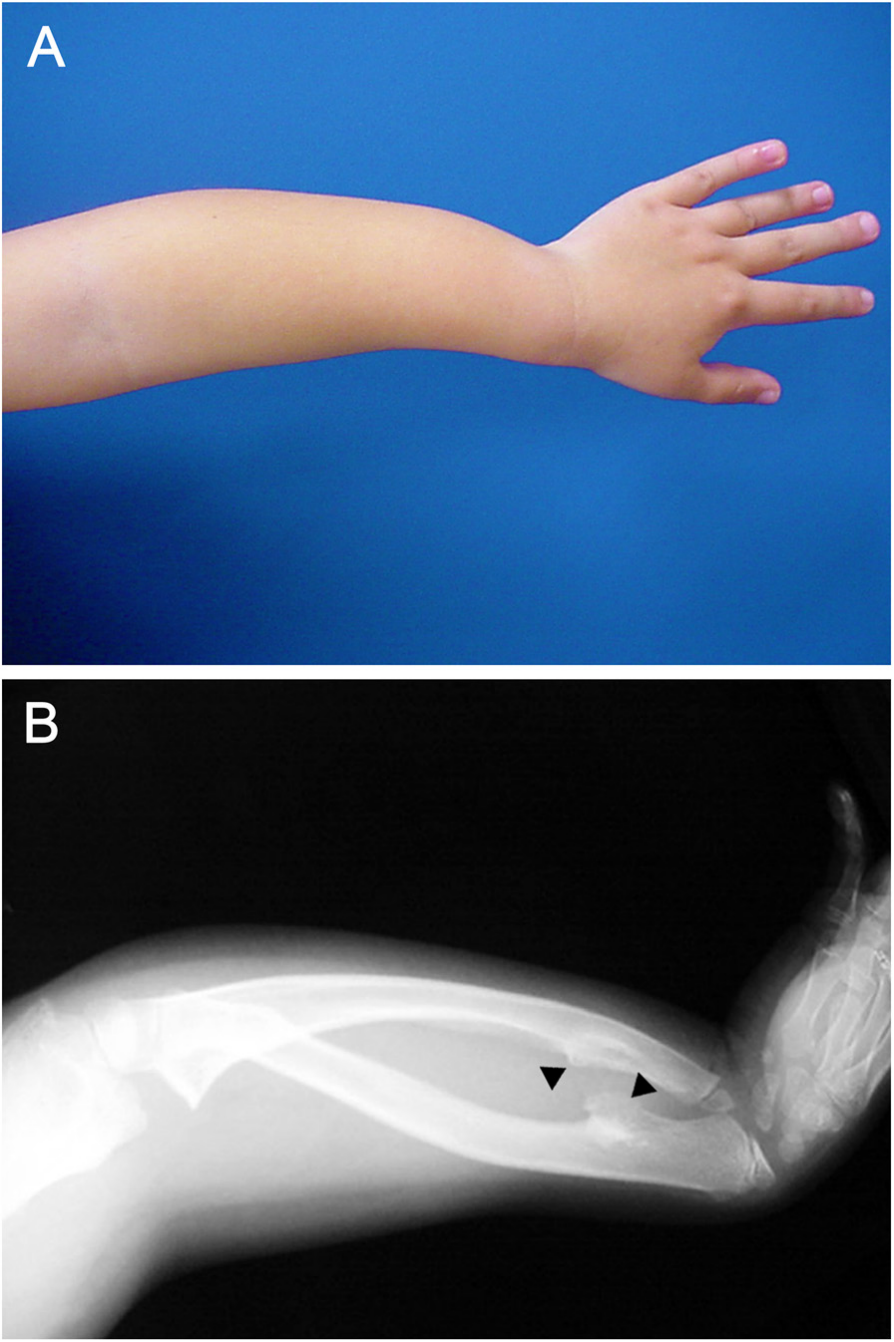
**Manifestations of multiple osteochondromas (MO) in a patient. A**, Photograph of a 7-year-old patient with MO demonstrates marked forearm deformities. **B**, In his radiograph, the most common type of deformity is seen: a combination of relative shortening of the ulna, bowing of the radius and ulna, increased ulnar tilt of the distal radial epiphysis, and ulnar deviation of the hand (Courtesy of Mikel San Julian, MD, Department of Orthopaedic Surgery and Traumatology, University Clinic of Navarra, Pamplona, Spain).

Heparan sulphate (HS) are glycosaminoglycans, heavily sulphated linear polysaccharides, that are present in all type of cells. Once they become attached to a core protein they form proteoglycans. The biosynthesis of HS take place in the Golgi apparatus and endoplasmic reticulum, where the elongation of glycosaminoglycan chains is maintained by type II glycosyltransferases encoded by the *EXOSTOSINs* genes, *EXT1* and *EXT2*[5]. Several genes are involved in the biosynthesis and degradation of HS, and mutations affecting the HS production have serious consequences. Abnormal accumulation of HS, due to its impaired degradation, causes mucopolysaccharidosis, a progressive disorder affecting mental and physical abilities, causing damage to various organs and leading to premature death. Patients with mucopolysaccharidosis often display skeletal abnormalities such as short stature or abnormal bone density [6,7]. Decreased levels of HS due to mutations in *EXT1* or *EXT2* also lead to a skeletal abnormality resulting in one of the most common benign bone tumours in young adults – osteochondroma [8]. The hereditary form of osteochondroma, multiple osteochondromas (MO; previously named multiple hereditary exostosis, MHE or hereditary multiple exostosis, HME), is a syndrome that is characterized by the development of multiple tumours (osteochondromas) at different sites of the endochondral skeleton [9]. MO is also associated with various other skeletal and non-skeletal phenotypes such as short stature, bone bowing (Figure 1), impingement of tendons, muscles or nerves as well as low bone density, lipid deposition within osteochondromas, pain and scarring [9-13].

Several mice models have been developed to study the role of *EXT1* or *EXT2* in bone and osteochondroma formation [14]. Zebrafish (*Danio rerio*) have also been shown to be a powerful animal model with morphological and developmental pathways comparable to those seen in humans [15]. We use zebrafish *dackel (dak)* mutants that carry a nonsense mutation in the *ext2*, gene which is 84.7% identical (at protein level) with human *EXT2*[16]. The *ext2*^*-/-*^ fish have been used as a model for MO. They have shown to mimic the cartilage phenotype (organization and behaviour) that is common to all models and the dental phenotype present in a number of patients but never described in mice [17-20].

In this study we show that bone development in the zebrafish *ext2*^*-/-*^ mutant is affected at two levels/stages in osteogenesis. Firstly development of the scaffold that is needed for osteoblasts to generate the bone is delayed/absent because chondrocytes fail to undergo terminal differentiation. Secondly, bone formation fails to progress from pre-osteoblasts towards osteoblasts and this change coexists with abnormal lipid depositions and premature adipocyte differentiation. Compounds stimulating fat-to-bone shift, GW9662 and purmorphamine, stimulate bone development in WT and *ext2* heterozygote but do not rescue the *ext2*-null bones. Reduced expression of *xbp1*, the master regulator of *osterix*, suggests that unfolded protein responses might play an important role in MO pathogenesis. Beside the “low bone-high fat phenotype”, the *ext2*-null fish also have a muscle phenotype, i.e. muscles are shorter and thicker, and therefore might have different mechanical properties. Bone bowing, weak muscles and muscle fatigue are often observed in MO patients. Based on our findings in the fish model we speculate that bone bowing may occur as a result of weaker “fat bones” being distorted by muscles (with different mechanical properties). In support of this concept misshaped clavicles and bowed Meckel’s are a very frequent phenomenon in the *ext2-null* mutant fish (data not shown).

## Materials and methods

### Animals

All experiments on zebrafish were performed in accordance with national and institutional guidelines for the care and use of laboratory animals. Zebrafish (*Danio rerio* H.) AB, golden and albino strains were used as wild type (WT) lines. Homozygote *dackel (dak, ext2*^*to273b*^*), knypek (kny, gpc*^*u34.8*^*), pinscher (pic, slc35b2*^*14MX*^*), hi307 (β 3gat3*^*hi307*^*)* and *hi954 (uxs1*^*hi954*^*)* mutants were obtained in natural crosses and staged according to Kimmel *et al*. [21]. The *dak* mutant was also kept in a *Tg*(*osteix:GFP)* background [22]. Unless stated otherwise, embryos were anaesthetized in tricane, fixed in 4% paraformaldehyde, dehydrated in a series of methanol dilutions and stored at -20°C.

Bones were stained with Alizarin red as described previously [17]. Lipid deposits were visualized with Oil red O as described by Li and co-authors [23].

### Drug treatment

Groups of 50 eggs were placed in a Petri dish with 20 ml E3 medium. Prior to treatment fish larvae were manually decorionated. GW9662 (Sigma) at concentration of 5-20 μM and/or purmorphamine (Calbiochem) at concentrations of 2,5-20 μM were added at 48, 60, 72, and 96 hours post fertilization (hpf) directly into E3 in which larvae were grown. 2-40 μM SB431542 (Tocris Biotrend), 0.2-4 μM dorsomorphin, 1-10 ng/ml TGF-β3 (Oncogene Sci.), or 10-2500 ng/ml BMP6 (a gift from Dr. K. Sampath, Curis, Cambridge, MA) were added into E3 from 48 hpf. For control, equal volume of DMSO (solvent) was added. In case of TGF-β3 and BMP6 activators, as a solvent and control, 4 mM HCl and 0.1% BSA were used. At 6 days post fertilization (dpf) fish were analysed for *osterix* expression (fish with transgenic *osterix:GPP* in the background) and for bone calcification (Alizarin red).

### In situ hybridization and immunohistochemistry

Whole mount mRNA *in situ* was done accordingly to Thiesse 2008 [24] using: *fabp11a, fabp11b* and *pparg* rybo-probes. For amplification of the probe templates following primers were used: *fabp11a_F* 5′-GATCAAATCTCAATTTACAGCTGTTG-3′, *fabp11a_R + T7* 5′-TAATACGACTCACTATAGGGTTCAAAGCACCATAAAGACTGATAAT-3′, *fabp11b_F* 5′-AACACTTTGTGCTATTATCTGTC-3′, *fabp11b_R + T7* 5′-TAATACGACTCACTATAGGGCCATCCGCAAGGCTCATAG-3′, *pparg_F2* 5′-TGCAGAGAACAGCGTTTCAT-3′ and *pparg_R1 + T7* 5′-TAATACGACTCACTATAGGGCACTTCGATGACCCCGTACT-3′. Whole mount immunostaining on zebrafish embryos was performed as described previously [17] using as a primary antibodies from the Developmental Studies Hybridoma Bank: anti-MF-20 for muscles and collagen II for cartilage, both in dilution of 1:250. For light microscopy, the anti-Digoxigenin-AP, Fab fragments (Roche) at 1:4000 or anti-mouse AP (Sigma) at 1:500 followed by BCIP/NBT (Sigma) were used to detect the signal. For confocal microscopy, Alexa 488 and 546 were used as the secondary antibody in dilution 1:200. Each experiment was repeated at least three times. Morphological evaluation was then performed by comparing of the *ext2* homozygote mutant with its normal counterpart.

### Quantitative RT-PCR

The expression levels of bone-, cartilage- and adipocyte-specific markers were determined by quantitative real time PCR. RNA extraction was performed as described by de Jong and colleagues with on-column DNase I digestion [25]. cDNA synthesis was performed as described previously [26]. Possible genomic contamination in the cDNA preparations was tested by PCR using *col1a2* primers and confirmed as negative. The primer sets were designed using Primer3 online program. The sequences of the qPCR primers are listed in Table 1. Unless stated otherwise, the primers were designed as such that the amplicons were 100–150 bp, spanning at least one intron. Tm was set at 60 ± 1°C. Quantitative real time PCR was carried out in BioRad iCycler system with SYBR Green SuperMix (BioRad), and was analysed with iCycler IQ (40 cycles, 1 min 95°C for denaturation and 1 min 60°C for annealing and elongation). All the samples were examined in duplicate or triplicate, and the expression of each marker was normalized to *slc25a5* level. *slc25a5* is one of a few house-keeping genes which, accordingly to our array data, is not differentially regulated in the *ext2*^*-/-*^ fish (unpublished).

**Table 1:** Sequences of primers used for quantitative PCR

**Primer name**	**Sequence 5′ → 3′**	**Lineage**	**Gene structure**
*adopql qPCR F1*	AACCTGGAAGAGATGGCAGA	A	
*adopql qPCR R1*	CAGGAAAGCCTCTTGGTCCT	A	
*cebpa qPCR F1*	CACAACAGCTCCAAGCAAGA	A	#
*cebpa qPCR R1*	AATCCATGTAGCCGTTCAGG	A	#
*cebpb qPCR F1*	TGTTCAGCCCGGACTTTATG	A	#
*cebpb qPCR R1*	AGTCTGGTACGGCAGGTACG	A	#
*col1a2 qPCR F2*	CTGGCATGAAGGGACACAG	B	
*col1a2 qPCR R2*	GGGGTTCCATTTGATCCAG	B	
*col10a1 qPCR F2*	CCTGTCTGGCTCATACCACA	C	
*col10a1 qPCR R2*	AAGGCCACCAGGAGAAGAAG	C	
*osteocalcin qPCR F1*	TGAGTGCTGCAGAATCTCCTAA	B	
*osteocalcin qPCR R1*	GTCAGGTCTCCAGGTGCAGT	B	
*osteopontin qPCR F1*	TGAAACAGATGAGAAGGAAGAGG	B	
*osteopontin qPCR R1*	GGGTAGCCCAAACTGTCTCC	B	
*osterix qPCR F2*	GCGTCGATTCTGGAGGAG	B	
*osterix qPCR R2*	AATCTCGGACTGGACTGGTG	B	
*pparg qPCR F1*	GGTTTCATTACGGCGTTCAC	A	
*pparg qPCR R1*	TGCGGCTCTTCTTGTGTATG	A	
*runx2a qPCR F1*	AACTTTCTGTGCTCGGTGCT	B, C	
*runx2a qPCR R1*	GTCATTTCCAGCCATTACCG	B, C	
*runx2b qPCR F2*	CAAACACCCAGACCCTCACT	B, C	
*runx2b qPCR R2*	GTATGACCATGGTGGGGAAG	B, C	
*scd1 qPCR F1*	GTGGCGAAATGTCATTCTGA	A	
*scd1 qPCR R1*	CCATACACGAAACACGCAAA	A	
*slc25a5 qPCR F1*	CCCCCATTGAGAGAGTCAAA	HK	
*slc25a5 qPCR R1*	CCTCTCCAGAACGACAGGAA	HK	
*sox9a qPCR F1*	GGAGCTCAGCAAAACTCTGG	C	
*sox9a qPCR R1*	AGTCGGGGTGATCTTTCTTG	C	
*srebp1c qPCR F1*	CCCCCAGCAGACTCTCTACA	A	
*srebp1c qPCR R1*	CGACAGACTCTGGATCGTCA	A	

A, adipocyte-specific gene; B, bone specific gene; C, cartilage specific gene; HK, house keeping gene. #, gene with one exon.

Unless stated otherwise, the primers were designed to construct amplicons of 100–150 bp, spanning at least one intron to avoid amplification from possible genomic DNA contaminant in the template.

### Lipid analysis

For Oil red O stain, 6 days old fish were anesthetized in tricane and fixed in 4% paraformaldehyde for 1–3 hours at room temperature prior to 10 minutes incubation with the dye. After staining, fish were washed twice in PBS and sorted by phenotype to homozygote mutant and siblings. Oil red O was extracted from a group of 10 phenotyped fish by over-night incubation in 100% methanol and quantified by measuring absorbance at 518 nm.

For TLC analysis, 6 days old fish were anaesthetized in tricane, sorted in groups of 20 fish. WT, siblings or homozygote mutants were ground with a plastic pestle in a mixture of chloroform:methanol (2:1, v/v) and incubated at room temperature for 15 minutes. To 1 ml of extract 300 μl of water was added. Samples were quickly vortexed at 2000 rpm for 5 minutes. Bottom phase was washed twice with 0.5 ml of water to be finally reduced in a speed-vacuum. Concentrated lipid extracts were spotted on a Silica gel 60 TLC plate (Merck). Plates were developed in a mixture of chloroform–ethanol–water–triethylamine (30:35:7:35, v/v/v/v), sprayed with primuline and viewed under ultraviolet light.

### Statistical analysis

Data are given as mean ± standard error of mean (SEM). One sample t-test for comparing column means to a hypothetical value or two samples unpaired Student’s test for comparison of two groups were used to determine statistical significance and described as * for p < 0.05, ** for p < 0.005 and *** for p < 0.001.

## Results

**Figure 2:**
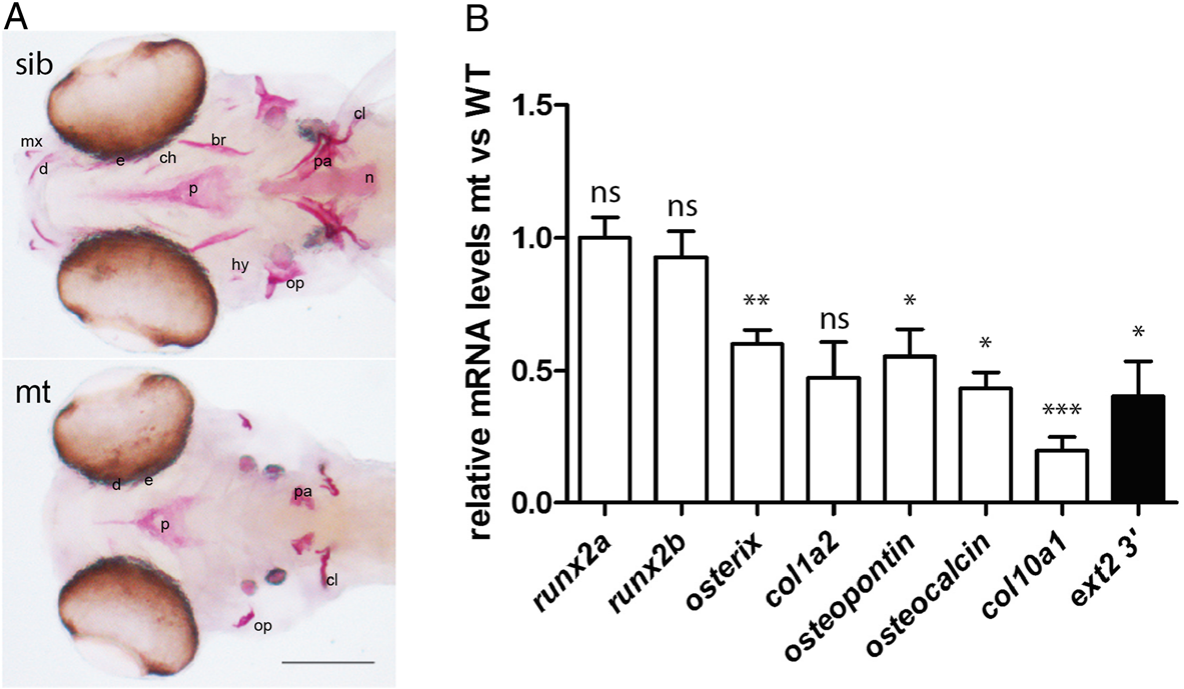
**Bone development is impaired in the *ext2***^***-/-***^**fish. A**, Alizarin red stain for ossification in the craniofacial skeleton at 6dpf. Dermal bones: dentary (d), maxilla (mx), parasphenoid (p), endopterygoid (e), branchiostegal rays (br), opercle (op), cleithrum (cl), cartilage bones: hyomandibula (hm), ceratohyal (ch). ceratobranchial 5/pharyngeal arch (pa); and notochord (n), scale = 0.1 mm; **B**, The relative change in the expression of bone markers at 5dpf was evaluated by real time PCR and analysed by delta-delta-Ct in the homozygote *ext2* mutants *vs*. wild type. The results represent an average from minimum four single embryos. Expression was normalized against *slc25a5*. Error bars indicate means with SEM. Expression of the *ext2* was given as an example of a gene that was approximately 2-folds down-regulated and this under-expression was of biological relevance.

Impaired bone development in the *ext2*^*-/-*^ fish has been described previously [17-19]. In order to identify at what step bone formation is affected and what mechanisms underlie changes in the *ext2* mutant, we examined the expression of various bone molecular markers in the *ext2*^*-/-*^ fish and compared it with its siblings (Figure 2).

### The importance of HS for pre-osteoblast differentiation

Our previous mRNA *in situ* analyses demonstrated normal expression patterns of *collagen2*, *sox9a*, and *chondromodulin* in the *ext2*^*-/-*^ fish, but did not give good estimates of the expression levels of these molecules [17,18]. In this work, using real time PCR, we confirm that in the homozygote *ext2* mutants, the expression levels of early skeletal markers such as *runx2* are maintained at wild-type-levels whereas late skeletal markers such as *osterix*, *collagen1a1, osteopontin* and *osteocalcin* are approximately 2-fold down-regulated and *collagen 10a1* shows even greater reduction (Figure 2B). Gene expression data indicate that HS are needed by chondrocytes for terminal differentiation for providing a scaffold for developing bone, and for maintenance of the osteochondroprogenitors/preosteoblasts to osteoblastic lineage.

### Bone loss coincides with elevated lipid levels, premature adipocyte differentiation and misshapen musculature in the *ext2*^*-/-*^ fish

Mesenchymal precursors can differentiate toward skeletal-forming cells (osteoblasts and/or chondroblasts) and/or other lineages such as myoblasts and adipocytes [27]. Although differentiation of each lineage is controlled by multiple factors including HS-dependent hedgehog, Wnt or BMP, a switch in the fate of single or multiple lineages can be trigged relatively easily. Thus, we assessed whether diminished bone development in the *ext2*^*-/-*^ fish is compensated with gain of other lineage(s).

**Figure 3:**
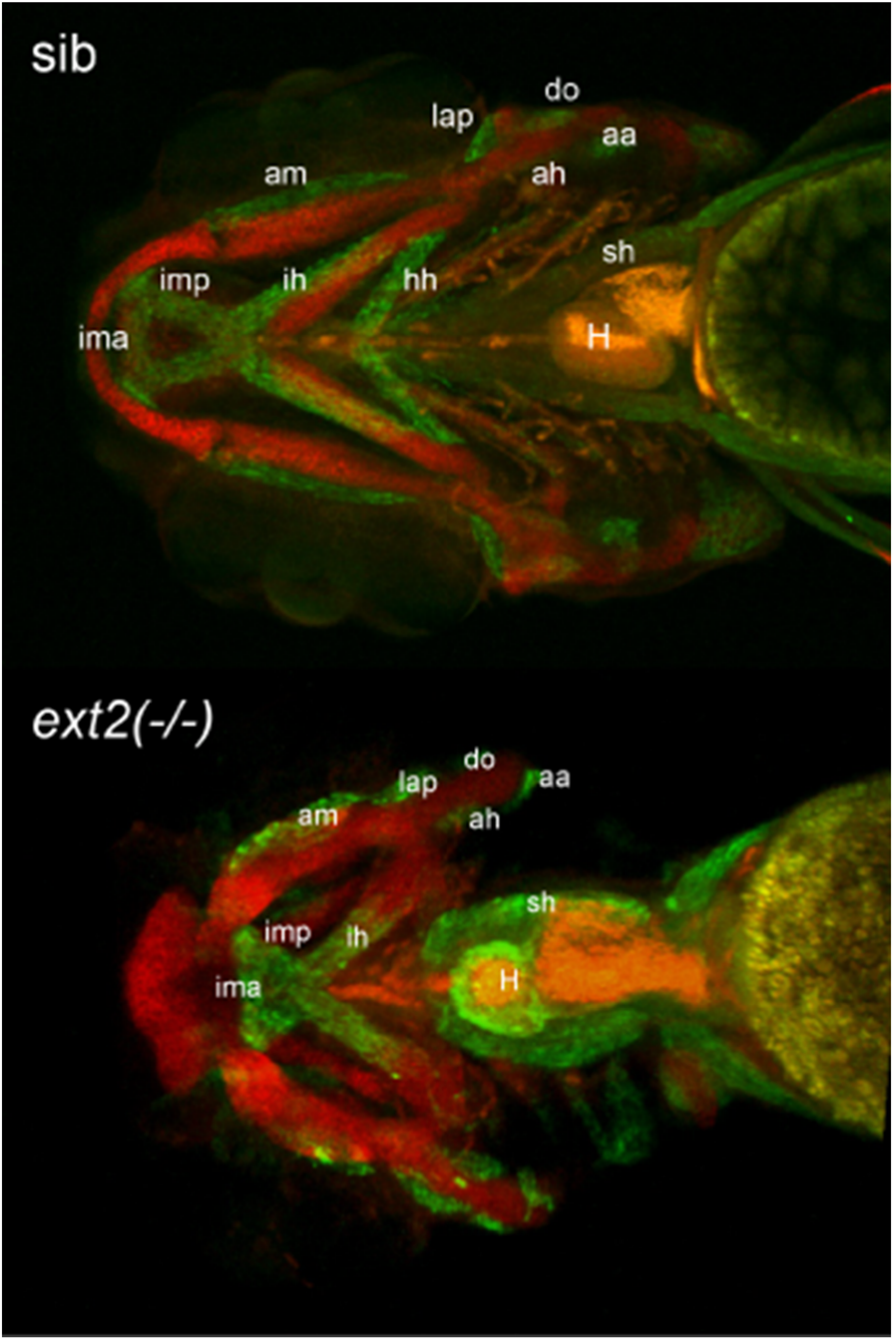
**Homozygous *ext2 *mutant displays musco-skeletal phenotype.** Whole mount immunolocalisation at 4dpf using MF-20 antibody for muscles (green) and collagen II for cartilage (red) shows thicker and shorter muscles fitting the malformed cartilaginous skeleton in the *ext2*^*-/-*^ fish. Muscles: *intermandibularis anterior (ima), intermandibularis posterior (imp), adductor mandibulae (am), interhyoideus (ih), hyohyoideus (hh), levator arcus palatine (lap), adductor hyoideus (ah), dilator operculi (do), sternohyoideus (sh) and adductor operculi (ao)*. Note missing hh muscle and, marked with a star, increased musculature around heart (H) in ext2-/-. Scale = 0.1 mm.

The whole mount MF-20-immunohistology revealed no obvious differences in the musculature between heterozygous *ext2* mutant and its wild type siblings (data not shown). However, the craniofacial muscles in the *ext2*^*-/-*^ fish were shorter, broader and fitted the misshapen cartilaginous skeleton (Figure 3). Moreover, some muscles such as the hh were absent, whereas extra deposition of muscles was observed around *ext2*^*-/-*^ heart (Figure 3, Table 2, Additional file 1).

**Table 2:** Cranial muscles in zebrafish head

				**3dpf**		**4dpf**		**5dpf**	
**Region**	**Time**	**Muscle:**		**Wild type**	***ext2 (-/-)***	**Wild type**	***ext2 (-/-)***	**Wild type**	***ext2 (-/-)***
M	62	Intermandibularis anterior	ima	x	x*	x	x	x	x
H	58	Interhyoideus	ih	x	x	x	x	x	x
B	62	Tranversus ventralis	tv	x	x	x	x	x	x
M	62	Intermandibularis posterior	imp	x	x	x	x	x	x
H	58	Hyohyoideus	hh	x	a	x	a	x	a
B	85	Rectus ventralis	rv	na	na	na	na	na	na
B	85	Rectus communis	rc	na	na	na	na	na	na
M	53	Adductor mandibulae	am	x	x	x	x	x	x
M	62	Levator arcus palatini	lap	x	x	x	x	x	x
M	62	Dilator operculi	do	x	x	x	x	x	x
H	68	Adductor hyoideus	ah	x	x	x	x	x	x
H	68	Adductor operculi	ao	x	x*	x	x*	x	x*
B	72	Dorsal pharyngeal wall	dpw	na	na	na	na	na	na
H	85	Levator operculi	lo	na	na	na	na	na	na
E	62	Inferior oblique	io	na	na	na	na	na	na
E	58	Inferior rectus	ir	na	na	na	na	na	na
E	58	Lateral rectus	lr	na	na	na	na	na	na
E	53	Medial rectus	mr	na	na	na	na	na	na
B	53	Sternohyoideus	sh	x	xx	x	xx	x	xx
E	58	Superior oblique	so	na	na	na	na	na	na
E	58	Superior rectus	sr	na	na	na	na	na	na
D	72	Protractor pectoralis	pp	a	x*	x	x	x	x

Muscles were scored based on the MF-20 immuno staining of myosin (see Additional file 1) as described in [28] and indicated as: present (x), absent (a), or not analysed (na). Asterisk indicate severely reduced muscles. Minimum seven fish were analysed in each group. B, Branchail arches; D, Dorsal posterior; E, Extraocular; H, Hyoid arch; and M, Manibular arch. Time – number of hours post fertilization at which a muscle become visible.

**Figure 4:**
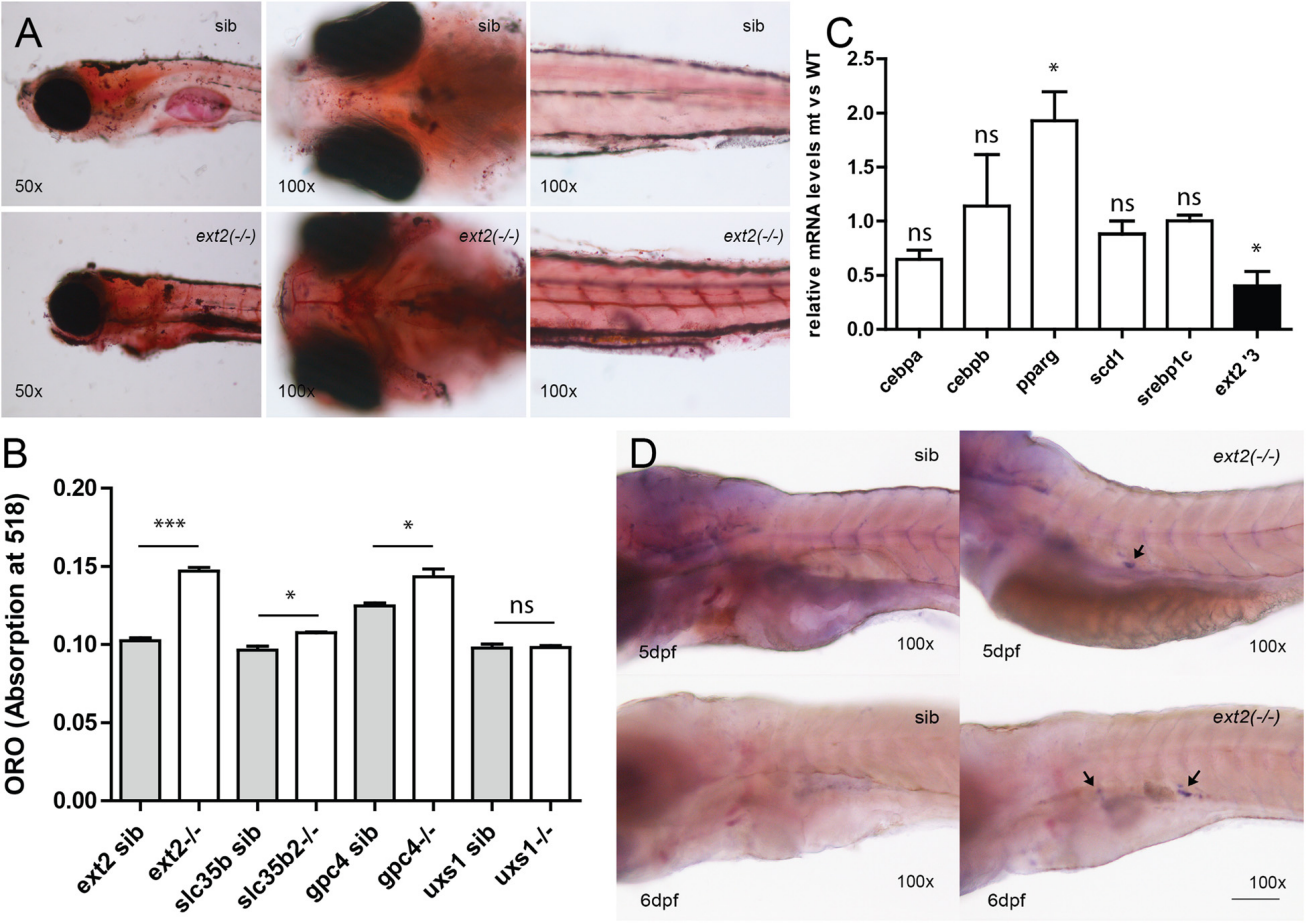
**Increased lipid levels in the *ext2***^***-/-***^**fish coincide with decreased bone formation. A**, Oil red O stain (ORO) in fish at 6dpf; **B**, the levels of lipids represented as absorbance of bounded ORO in 6 days old proteoglycan mutants: *ext2, slc35b2, gpc4* and *uxs1* (for more description see Additional file 2); **C**, the level of transcripts in 5-days old fish determined by quantitative PCR and normalised to *slc25a5* level. Primer sequences are given in Table 1; **D**, whole mount *fabp11a* mRNA *in situ* hybridisation showing prematurely developing adipocytes in the belly of the *ext2*^*-/-*^ fish. Error bars represent means with SEM.

Oil red O, a stain for neutral triglycerides, lipids and some lipoproteins, highlighted blood vessels, heart, tectum, guts, swim bladder and the remains of yolk in all fish (Figure 4A). In the *ext2*^*-/-*^ fish, the staining was intense and abnormally high lipid accumulation was observed. Especially, deposits in the vasculature were more pronounced (Figure 4A). Staining at the position of missing bones could be observed in some larvae. Significantly stronger (P < 0.001) Oil red O stain in the *ext2*^*-/-*^ fish coincided with over two fold overexpression of *pparg* (Figure 4)*.* Other adipogenic markers such as *cebp*, *srebp1c* and *scd1* were expressed at levels similar to wild type (Figure 4C). Despite intense staining, abnormal accumulation of lipids and overexpression of *pparg*, TLC analysis of lipid extracts did not reveal any changes in the profiles from wild type and *ext2*^*-/-*^ fish (data not shown).

Zebrafish adipocytes start to form by 8dpf and only upon feeding [29]. Interestingly, in the *ext2*^*-/-*^ fish, the mRNA *in situ* hybridization showed that *fabp11a-*expressing cells are present in unfed larvae already at 5dpf (Figure 4D).

### Bone-to-fat switch in proteoglycan mutants

Observing a disturbance in the differentiation of mesenchymal cell lineages, we wonder if this is specific to the *ext2* mutant, or to proteoglycan deficiencies in common. Using a panel of mutants described in previous studies [18,20], we found that the *hi954* (*uxs1*) mutant lacking various proteoglycans and with a mild bone phenotype did not show any alteration in lipid deposition as judged by Oil red O (Figure 4B). Significantly increased (P < 0.005) levels of lipids were detected in the *knypek (kny, gpc4*^*-/-*^*)* mutant, which lacks only a portion of HS and has a mild bone phenotype (Figure 4B and Additional file 2) [18,20]. Interestingly, the *pinscher (pic/slc35b2)* mutant, which fails to sulphate different molecules (including HS) and has a stronger bone phenotype [17,18], only showed a very small, but statistically significant increase in lipid levels (P < 0.05).

### Can PPARG inhibition rescue bone formation in the *ext2*^*-/-*^ homozygote mutant?

**Figure 5:**
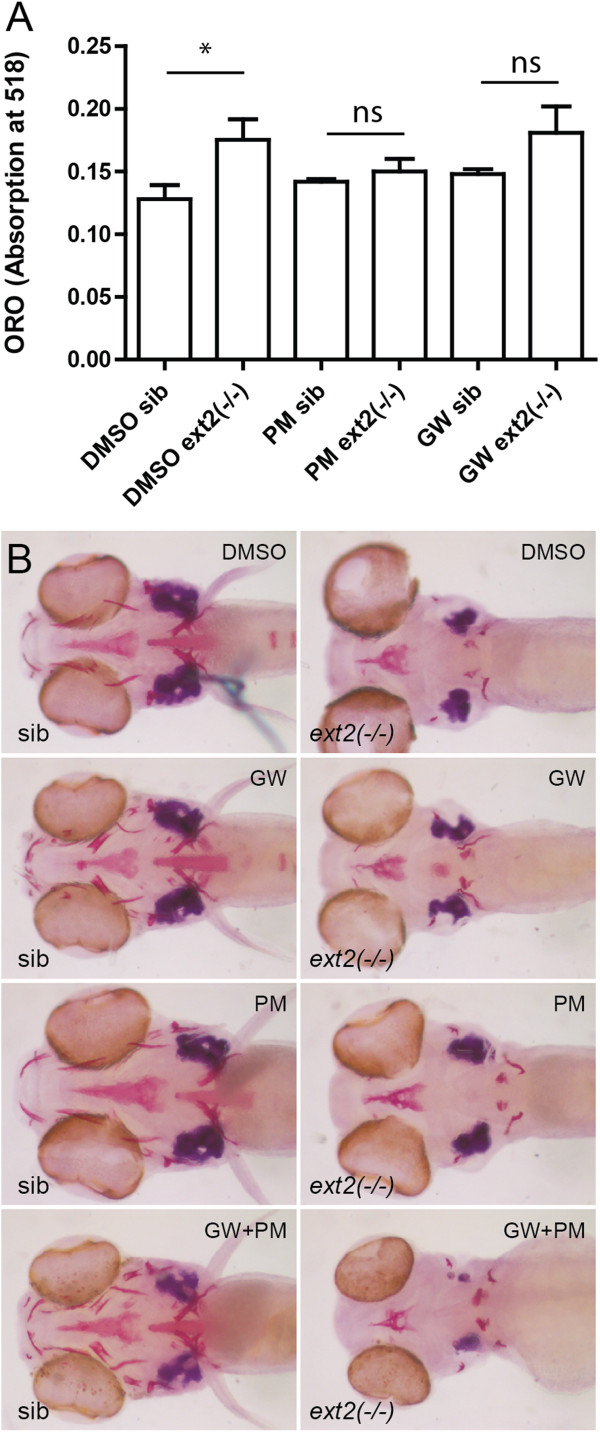
**Bone and lipid phenotypes in the *ext2***^***-/-***^**fish are partially rescued by inhibition of PPAR or activation of hedgehog signalling. A**, Oil red O (ORO) stain for lipids in fish at 6dpf; **B**, Alizarin red stain for bones in fish at 6dpf. GW9662 (GW) and purmorphamine (PM) were added into fish water by 60hpf and fish were raised to 6dpf. Minimum 10 fish were scored from each group and experiment was repeated with similar results at least three times. Error bars represent means with SEM.

Several drugs are known to affect lipid metabolism and influence the bone-to-fat balance. Although it is unlikely to expect a strong effect on total lipid levels in the early stages of zebrafish development where the majority of lipids come from yolk, application of GW9662, the antagonist of PPARG, was shown to enhance bone differentiation in zebrafish larvae [30]. As expected, we found that treatment with 15 μM GW9662 added at 60hpf did not have any significant effect on lipid levels (Figure 5A) but did enhance formation of cartilage and dermal bones in wild type and in the *ext2* heterozygous mutant (Figure 5B). In the *ext2*^*-/-*^ fish, with the same treatment, enhanced GFP expression was noted in *tg*(*osterix:gfp)* larvae (data not shown) with improved ossification of the previously existing bones. Bones that normally do not develop in *ext2*^*-/-*^ mutants, responded only partially to the treatment with rescue and stimulated ossification being observed in only some of the dermal bones; the *ext2*^*-/-*^-cartilage bones were not rescued by this treatment (Figure 5B). Similar effects were seen upon ≥7.5 μM purmorphamine treatment, which should stimulate a fat-to-bone switch by activating hedgehog signalling (Figure 5). Furthermore, we tested involvement of other signaling pathways (HS-dependent) which stimulate bone-to-fat change. Treatment with BMP6 (an activator of BMP pathway) or dorsomorphine (an inhibitor of BMP) did not show significant effect at any time point on the craniofacial *ext2*^*-/-*^ bones and TGF-β activator (TGF-β3 ligand) only partially stimulated dermal bones (data not shown).

### Is Ira1/Xbp1 pathway involved in the bone/lipid phenotype of the *ext2-/-* fish?

Recently, *Xbp1* was shown to regulate osteoblast differentiation in a *Runx2* independent manner [31]. Since in the *ext2*^*-/-*^ fish the levels of *runx2* transcript were normal while *osterix* levels were reduced, we wondered if the unfolded protein response is affected by the lack of HS. We found that heterozygotes maintained WT-levels of *ern1* and *xpb1*. In the *ext2*^-/-^ mutant, the expression of *ern1* was only slightly downregulated (2^∆∆^Ct *ext2*^*-/-*^/WT = 0,71), but the expression of its downstream target, the *xbp1*, was reduced to 0,64.

## Discussion

Abnormal lipid deposition coinciding with impaired bone formation is not common to all types of proteoglycan deficiencies (see Additional file 2). *b3gat3-* and *uxs1*-homozygote mutants, that are upstream of *ext2* in the biosynthesis pathway and lack heparan and chondroitin sulphates, have a very mild bone phenotype and do not show increased lipid deposition (this work and data not shown). Interestingly, the *fam20b* and *xylt1* mutants downstream of *uxs1* and upstream of *b3gat3* and *ext2* were shown to have enhanced bone ossification [32]. Unfortunately nothing is known about *fam20b* and *xylt1* lipid metabolism. The *ext2*^*-/-*^ and *gpc4*^*-/-*^, two mutants with reduced HS-levels only, have high lipid content; but only the *ext2* mutants have severely reduced bone formation, while the *gpc4*-null fish have very mild bone impairment. The *slc35b2* homozygote mutant, which has diminished levels of all sulphated proteoglycans, has an even more severe bone phenotype than the *ext2*^*-/-*^ fish and show only very mild enhancement of lipid deposition. Why different proteoglycan deficiencies have such different effects on bone and lipid metabolisms is not clear. Holmborn and coauthors [33] showed that, in the *ext2* homozygote mutant, the remaining HS are over-sulphated which changes their properties (i.e. increase occurrence of protein-interacting domains). Although, heparin, a highly sulphated glycosaminoglycan and a potent anticoagulant, which is often used in clinical practice, negatively affects bone density and is known to increase lipid deposition in sera, the role of over-sulphation of (proteo-)glycans would need to be confirmed.

Craniofacial skeletal development in zebrafish is of mixed origin being derived from cranial neural crest and/or mesoderm [34]. The presence of one functional copy of the *ext2* gene is sufficient for the maintenance of normal differentiation of chondrocytes, osteoblasts and other mesenchyme-derived cells. Reduction in HS-levels in the *ext2*^*-/-*^ larvae clearly affects skeletal development. Loss of bones cannot be linked specifically to one type of precursor cell as both neural crest- and mesoderm-derived structures are affected. Despite their origin, two populations of osteoblasts with different sensitivity to hedgehog signalling have been described in zebrafish [35]. As no defects in the hedgehog signalling were found in the craniofacial skeleton of the *ext2*^*-/-*^ fish, it is unlikely that bone defects could be linked to a specific type of hedgehog-sensitive osteoblast. However, it is possible that there are multiple types of osteoblasts existing in fish, differing in their sensitivity for HS.

Bone homeostasis depends on the balance between osteoblastic and osteoclastic activity. Lipids are known to attract osteoclasts while suppressing osteoblastogenesis (for review see [36]). Unfortunately, we were not able to test this in zebrafish as the first osteoclasts develop by 16 dpf, beyond the time of premature death of the *ext2*^*-/-*^ fish. Nevertheless, observations from patient material suggest that indeed both osteoblasts and osteoclasts are affected by HS-deficiencies [10,26] or by HS abnormal accumulation [7] and, in both cases, bone mineral density is altered. Osteoblasts and adipocytes might not be the only lineages affected by imbalanced HS. *EXT1-null* embryonic stem cells also appear to have impaired differentiation hematopoietic lineages [37], while osteochondromas exhibit impaired vascularisation [38].

Fatty acids, when not stored in adipocytes, accumulate into the circulation [39]. Although premature adipocyte-like cells were detected in the *ext2*^*-/-*^ fish it is unlikely that they would be able to store all the lipids as cytoplasmic droplets. Therefore, Oil red O stain in vasculature could reflect only a surplus of fatty acids/lipids. However, it is also possible that mutation in the *ext2* gene leads to an abnormal intravascular accumulation of lipids. The changes in bones and fat that we have described in fish were a characteristic of an organism homozygous for a mutation in the *ext2* gene in all cells. Since MO patients are mostly heterozygous for a mutation in *EXT* they should have very mild (if any) systemic phenotype. However, if findings from this fish model are true for humans, strong focal changes should be expected at the site where loss of heterozygosity/haplo-insufficiency occured. Not much is known about lipid metabolism in patients with MO. Lemos and co-authors [10] reported lower bone mineral density of femoral neck and lumbar spine in MO patients near osteochondromas. In addition, single reports described deposition of fat within the cartilaginous cap of osteochondromas [11] and development of lipoma, a benign bone tumour, or fat-pads in association with osteochondromas [40,41]. These finding might have been coincidental in MO but increased lipid levels often remain asymptomatic. In light of our findings in the fish model on the bone-fat imbalance the status of lipids in human MO seems worth investigating.

Humans, mice and fish with MO are often short in stature and have bowed bones. Recently, Jones and co-authors [42] demonstrated that osteochondroma growing on account of deranged bone growth is apparent only in some individuals and other mechanisms must contribute to the short bone phenotype. Also bone bowing does not always require osteochondroma formation to generate the observed anatomical changes (K. Jones, University of Utah School of Medicine, personal communication). The presence of muscle phenotype needs to be confirmed in non-fish MO. Further work will show how (if) muscles with different mechanical properties contribute to the formation of shorter and bowed bones in patients.

## Conclusions

Our data indicated that HS have multiple functions during endochondral bone development. First of all, HS are required for terminal differentiation of the cartilaginous template and consecutive formation of a scaffold that is needed for further bone development. Secondly, normal expression of *runx2* and impaired expression of *osterix* in the *ext2*^*-/-*^ fish indicated that HS are required by osteoblast precursors for their further differentiation within the osteoblastic lineage. Furthermore, the increased lipid deposition in the *ext2*^*-/-*^ fish suggest that HS are involved in determining the cell lineage when mesenchymal precursor cell differentiates into bones and/or fat. PCR analyses confirm the increase in the expression of lipid markers and down-regulation of early skeletal markers. It still remains to be established how HS are involved in this shift, but lower expression of *xbp1,* a master regulator of *osterix,* suggests that HS affect the unfolded protein response, a pathway which is known to control bone formation and lipid metabolism.

### Supporting data

The data sets supporting the results of this article are included within the article and its additional files.

## Abbreviations

AP: Alkaline phosphatase; b3gat3: *Beta-1,3-glucuronyltransferase 3*; BCIP/NBT: 5-bromo-4-chloro-3-indolyl-phosphate/nitro blue tetrazolium; BMP: Bone morphogenetic proteins; BSA: Bovine serum albumine; dak: *dackel*; Dpf: Days post fertilization; ext1: *exostosin 1*; ext2: *exostosin 2*; HS: Heparan sulphates; HSPG: Heparan sulphate proteoglycan; Hpf: Hours post fertilization; kny: *knypek*; GFP: Green fluorescence protein; MO: Multiple osteochondromas; pic: *pinscher*; pparg: *peroxisome proliferator-activated receptor gamma*; runx2: *runt-related transcription factor 2*; slc35b2: *transport of adenosine 3′-phospho 5′- phosphosulfate (PAPS)*; TCL: Thin layer chromatography; TGF: Transforming/tumour growth factor; uxs1: *UDP-glucuronic acid decarboxylase 1*; xbp1: *x-box binding protein 1*.

## Competing interests

All authors declare that they have no competing interests.

## Authors’ contributions

MIW, CEdA, KWFS, ZZ and PCWH designed this study, analyzed interpreted data; MIW, CEdA and KWFS collected data and generated figures. All authors were involved in writing the paper and had final approval of the submitted and published versions.

## Supplementary Material

Additional file 1 **Muscle phenotype in the *ext2***^***-/-***^**fish.** Muscles were detected with MF-20 antibody. Scale = 0.1 mm. Click here for file

Additional file 2 **Information about affected proteoglycans and the bone- and fat phenotypes of mutants used in this study.** Proteoglycans (PGs), heparan sulphate (HS), dermatan sulphate (DS), chondroitin sulphate (CS). keratan sulphate (KS) proteoglycans are the forth group of proteoglycan that is defected the *slc35b2*^*-/-*^ mutant. Click here for file
