# Supplementary figures and images for: Possible effects of EXT2 on mesenchymal differentiation - lessons from the zebrafish
Source: Orphanet J Rare Dis. 2014 Mar 14;9:35. doi: 10.1186/1750-1172-9-35 (PMC4004154; doi:10.1186/1750-1172-9-35)

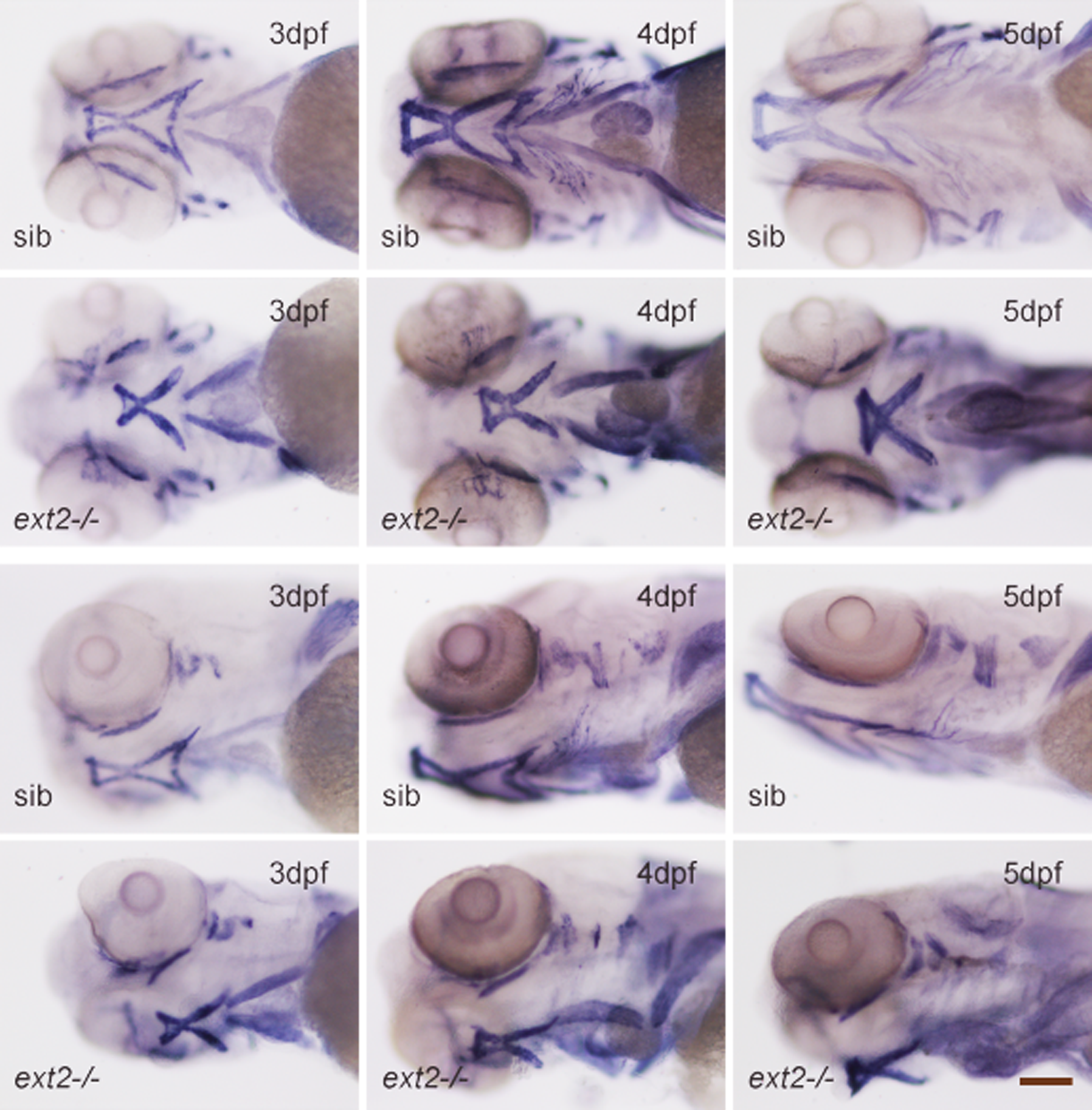

Supplement: Additional file 1 — Muscle phenotype in the ext2-/-fish. Muscles were detected with MF-20 antibody. Scale = 0.1 mm. [file 1750-1172-9-35-S1.tiff]
