# Supplementary material for: Possible effects of EXT2 on mesenchymal differentiation - lessons from the zebrafish
Source: Orphanet J Rare Dis. 2014 Mar 14;9:35. doi: 10.1186/1750-1172-9-35 (PMC4004154; doi:10.1186/1750-1172-9-35)
Supplement: Additional file 2 — Information about affected proteoglycans and the bone- and fat phenotypes of mutants used in this study. Proteoglycans (PGs), heparan sulphate (HS), dermatan sulphate (DS), chondroitin sulphate (CS). keratan sulphate (KS) proteoglycans are the forth group of proteoglycan that is defected the slc35b2-/- mutant. [file 1750-1172-9-35-S2.doc]

Additional file 2. Information about affected proteoglycans and the bone- and fat phenotypes of mutants used in this study. Proteoglycans (PGs), heparan sulphate (HS), dermatan sulphate (DS), chondroitin sulphate (CS). keratan sulphate (KS) proteoglycans are the forth group of proteoglycan that is defected the *slc35b2^-/-^* mutant.

*____________________________________________________________________________________________________________________*Gene name Affected proteoglycans Bone phenotype Fat phenotype

*____________________________________________________________________________________________________________________*

*PAPS transporter 1* (*slc35b2)* loss of sulphation in all PGs most severe bone loss slightly enhanced lipid deposition

*UDP-glucuronate decarboxylase* *(uxs1)* loss of HS-, CS- and DSPGs subtle bone phenotype no fat phenotype

*glycosaminoglycan xylosylkinase (fam20b)* loss of HS-, CS- and DSPGs enhanced bone ossification not analysed

*xylosyltransferase 1* (*xylt1)* loss of HS-, CS- and DSPGs enhanced bone ossification not analysed

*beta-1,3-glucuronyltransferase 3 (b3gat3)* loss of HS-, CS- and DSPGs subtle bone phenotype not analysed

*exostosin 2 (ext2)*  short HSPGs (more CSPGs) severe bone reduction enhanced lipid deposition

*glypican 4 (gpc4)* some HSPGs only mild bone reduction enhanced lipid deposition

___________________________________________________________________________________________________________________
